# Icariin promotes bone marrow mesenchymal stem cells osteogenic differentiation via the mTOR/autophagy pathway to improve ketogenic diet-associated osteoporosis

**DOI:** 10.1186/s13018-024-04529-x

**Published:** 2024-02-07

**Authors:** Wei Liu, Shouyu Xiang, Yingcong Wu, Dinghao Zhang, Chuhai Xie, Hailan Hu, Qi Liu

**Affiliations:** Division of Spinal Surgery, Department of Orthopedics, The Second Affiliated Hospital of Guangzhou Medical University, No.250, Changgang East Road, Guangzhou, 510260 Guangdong China

**Keywords:** Icariin, mTOR, Ketogenic diet, Osteoporosis, Autophagy

## Abstract

**Background:**

Icariin, a traditional Chinese medicine, has demonstrated anti-osteoporotic properties in ovariectomized mice. However, its effectiveness in preventing bone loss induced by ketogenic diet (KD), which mimics osteoporosis in human, remains unexplored. This study aims to investigate icariin’s impact on KD-induced bone loss in mice.

**Methods:**

Thirty mice were divided into: sham, KD, and KD + icariin groups. Post a 12-week intervention, evaluation including bone microstructures, serum concentrations of tartrate-resistant acid phosphatase (TRAP) and bone-specific alkaline phosphatase (ALP), and femoral tissue expression levels of osteocalcin (OCN) and TRAP. The expression levels of mammalian target of rapamycin (mTOR), ALP, peroxisome proliferator-activated receptor gamma (PPAR-γ), phosphorylated mTOR (p-mTOR), and the autophagy adaptor protein (p62) were also analyzed. Alizarin granule deposition and cellular ALP levels were measured following the induction of bone marrow mesenchymal stem cells (BMSCs) into osteogenesis.

**Results:**

The study found that KD significantly impaired BMSCs' osteogenic differentiation, leading to bone loss. Icariin notably increased bone mass, stimulated osteogenesis, and reduced cancellous bone loss. In the KD + icariin group, measures such as bone tissue density (TMD), bone volume fraction (BV/TV), trabecular number (Tb.N), and trabecular thickness (Tb.Th) were significantly higher than in the KD group. Additionally, bone trabecular separation (Tb.Sp) was markedly lower in the KD + icariin group. Moreover, icariin increased OCN and ALP levels while suppressing PPAR-γ, TRAP, p62, and p-mTOR. In cellular studies, icariin encouraged osteogenic development in BMSCs under KD conditions.

**Conclusions:**

Icariin effectively counteracts bone thinning and improves bone microstructure. Its mechanism likely involves stimulating BMSCs osteogenic differentiation and inhibiting bone resorption, potentially through mTOR downregulation. These findings suggest icariin's potential as an alternative treatment for KD-induced bone loss.

## Introduction

Osteoporosis, a widespread bone disease, is marked by microstructural deterioration and reduced bone mass, affecting over 200 million individuals globally. This condition heightens the risk of fractures and poses an increasing economic burden as the population ages [1, 2]. Osteoporosis is a chronic disorder of bone metabolism influenced by factors such as age, hormone levels, medications, physical activity, and diet, it disrupts bone metabolism [3]. Migliorini et al. [4–8] have done a series of researches on osteoporosis and its treatment. Currently, treatments primarily aim to curb bone resorption and promote bone growth, as resorption typically exceeds production in osteoporosis. Clinical treatments often including denosumab and bisphosphonates [9]; however, these require long-term administration and pose risks of adverse effects [10]. In contrast, traditional Chinese medicine (TCM) offers a viable long-term alternative with fewer side effects, with agents such as icariin, naringin, and neofururin showing promise in combatting osteoporosis [11].

Basic research in osteoporosis often focuses on postmenopausal osteoporosis, glucocorticoid-induced osteoporosis, and senile osteoporosis as models for animal studies [12]. Recent discourse highlights the significantly role of diet and lifestyle in osteoporosis development. Studies indicate that obesity and a high-fat diet lead to compromised osteogenic differentiation, increased osteoclast activity, and notably lower bone density in mice [13]. Diverging from typical high-fat diet, the ketogenic diet (KD) entails very low carbohydrate intake and is predominantly fat-based. Initially used for epilepsy treatment in the 1920s [14], KD has since been applied in managing cancer, cardiovascular disease, and endocrine disorders [15]. KD’s adverse effects include severe bone loss and damaging bone structure. Research shows that KD impairs the bone biomechanical strength and hinders osteogenic differentiation of bone marrow stem cells (BMSCs) [14, 16].

Autophagy, the process by which self-renew by degrading damaged organelles and proteins, plays a crucial role in the development and progression of diseases related to metabolic dysregulation, including cancer, osteoporosis, and neurodegenerative disorders. mTOR is identified as a pivotal regulator of cell growth, proliferation, survival, and a central element in signaling pathways governing [17]. The previous research suggests that the high-fat diet may inhibit the differentiation of BMSCs into osteoblasts by modulating autophagy, potentially contributing to the onset of osteoporosis [18].

Icariin, an active flavone glycoside produced from Epimedium plants, exhibits a broad spectrum of pharmacological effects, encompassing immunological, anti-inflammatory, anti-tumor, cardiovascular, and neuroprotective properties [19]. Recent studies have highlighted icariin’s potent anti-osteoporotic effects, evidenced by increased bone formation and reduced bone resorption [20]. In ovariectomized rats, icariin notably delays the onset of osteoporosis and accelerates the healing of osteopoporotic fractures [21]. Research by Liang et al. demonstrated that icariin promotes osteogenic differentiation of BMSCs and inhibits bone loss in mice by activating autophagy [22]. Furthermore, Tang et al. showed that icariin regulates chondrocyte autophagy through the PI3K/AKT/mTOR pathway, elucidating its impact on mTOR [23]. However, the efficacy and specific mechanism of icariin in mitigating bone loss induced by KD remains to be elucidated.

This study aimed to assess icariin’s impact on KD-induced osteoporosis and to identify underlying pathways. We proposed that icariin: (1) upregulates mTOR, facilitates cellular autophagy, and stimulates osteogenic differentiation of BMSCs; and (2) enhances osteoblast activity, reduces osteoclast activity, and fosters bone remodeling.

## Materials and methods

### Collection of animal specimens and icariin treatments

Thirty female C57BL/6 J mice, aged 8 weeks, were equally divided into three groups: sham (*n* = 10), KD (*n* = 10), and KD + icariin (*n* = 10). The KD and KD + icariin groups consumed a ketogenic diet (Jielikang, Shenzhen, China) with a 1:3 ratio of carbs to lipids. The sham group was provided standard diet by the Lab Animal Center at Southern Medical University. All groups had unrestricted access to food and water. The diet met AIN-93 guidelines [24]. The KD group did not receive icariin, whereas the KD + icariin group was administered icariin intraperitoneally at a dose of 50 mg/kg daily. Housing conditions including a wire cage, a 12-h light cycle, 25 °C temperature, and 48% humidity. After 12 weeks, mice were anesthetized 1% sodium pentobarbital. Blood samples were then collected via intra-puncture, with serum separated by centrifugation 1000 g and subsequently stored at -80 °C in liquid nitrogen. The right femur was used for Western blot analysis and BMSCs' differentiation culture, while the left femur was underwent micro-CT scanning, histological, and immunohistochemical analysis.

### Measurement of bone turnover markers, blood glucose, blood ketone, and body weight

For a period of 12 weeks, the body weight of each group was measured every 4 weeks. The Yicheng Blood Ketone Meter T-1 (Sentest Inc., China) and JPS-5 (Leapon Inc., China) were employed to assess blood ketone and glucose levels by accessing the tail veins. Every 4 weeks, glucose and ketone levels were alternately measured in only four of the total mice to limit potential damage. The amounts of tartrate-resistant acid phosphatase (TRAP) and bone-specific alkaline phosphatase (ALP) were quantified using an ELISA kit (CUSABIO, Wuhan, China).

### Analysis of bone microstructures

The left femur was utilized for microstructure of the bone. Scanning of the distal metaphysis was performed using a high-resolution micro-CT system (CT 80, Scanco Medical AG, Switzerland), featuring an isotropic voxel size of 10 m, 55-kV tube voltage, and 145-A tube current. Micro-CT system software enabled the analysis of trabecular structure and facilitated 3D reconstruction. Analysis and reconstruction focused primarily on the distal metaphysis, commencing 2.0 mm from the proximal tip of the primary spongiosa. Parameters such as trabecular number (Tb.N), trabecular thickness (Tb.Th), trabecular separation (Tb.Sp), bone volume/tissue volume (BV/TV), and tissue mineral density (TMD) were assessed.

### Immunohistochemical and histological staining

The left distal femur was embedded in olefins for subsequent histological and immunohistochemical analysis after completing the microstructure of bone investigation. Hematoxylin and eosin staining was used to examine the morphology of trabecular bone tissue. Additionally, osteocalcin staining (OCN) (ab41928; Abcam Biotechnology, MA, USA; the dilution ratio is 1:100) was utilized to assess the activities of osteoblast, and TRAP staining (Sigma-Aldrich, St. Louis, MO, USA) was used to assess the osteoclast activity. In semiquantitative immunohistochemistry analysis, we captured four distinct images at 400 × magnification using Image Pro Plus software (Media Cybernetics, MD, USA) for each slide. The number of positive cells was quantified by counting them on each bone surface (BS), and staining intensity was evaluated using the integrated optical density (IOD/area, mean density) of each positive cell area.

### Western blot

Total protein from the proximal half of the right femur was extracted using the RIPA protein extraction reagent (Boster Biological Technology, Wuhan, China), following the manufacturer's protocol. The proteins were separated using SDS-PAGE on a 10% gel and subsequently transferred to a nitrocellulose membrane. Non-specific binding sites were blocked by incubating with 5% blocking buffer (5% bovine serum albumin in Tris-buffered saline with 0.1% Tween 20 (TBS-T) for 1 h at room temperature). The membrane was then incubated overnight at 4 °C with the primary antibodies of OCN, PPAR-γ, p62, mTOR, and p-mTOR (all at a 1:1000 dilution ratio, Abcam Biotechnology, MA, USA). This was followed by a 2-h room temperature incubation with an HRP-conjugated secondary antibody (Boster Biological Technology, Wuhan, China), diluted at 1:1000. Post-incubation, the membrane was exposed for 5 min to enhanced chemiluminescence for immunoreactive protein visualization. Semiquantitative analysis was performed using ImageJ software. Protein expression normalization and total protein quantification were facilitated using anti-GAPDH (Abcam Biotechnology, MA, USA).

### Osteogenic potential

The right distal femur was harvested and briefly immersed in 70% ethanol before being preserved in sterile PBS (1% penicillin streptomycin and 0.1% fungizone). The epiphyses of each bone were removed, and the bone marrow was flushed from the shaft using a syringe, then collected in medium supplemented with 10% FBS, 1% penicillin streptomycin, 0.1% fungizone, L-glutamine, and nucleosides. After filtering, the cell suspension was combined with primary medium and centrifuged (800 rpm/min for 3 min), resulting in a cell concentration of 5 × 10^6^ cells, which were then cultured in a 6-cm tissue culture plate. On day 7, adherent cells received secondary medium (consisting of primary medium enriched with 50 g/ml L-ascorbic acid, 10-mM β-glycerophosphate, and 100-nm dexamethasone) to induce osteoblast differentiation from BMSCs. Nonadherent cells were removed by gentle aspiration. The medium was subsequently refreshed every 2 days for the following 2 weeks. After 7 days of induction, the ALP activity was measured in each cell after 7 days of induction, and at 14 days, the calcified nodules were stained with alizarin red.

### Analytical statistics

Using SPSS 20.0 for data analysis, repeated measurements and Student–Newman–Keuls (SNK) post hoc tests were conducted to determine the significance of changes in body weight, blood glucose, and blood ketones. One-way analysis of variance (one-way ANOVA) was applied for assessing serum markers, bone microstructures, immunohistochemistry, and Western blots, followed by Fisher's least significant difference (LSD) post hoc analysis. The threshold for statistical significance was *P* < 0.05.

## Results

### Icariin's impacts on blood ketone, blood glucose, and body weight

**Fig. 1:**
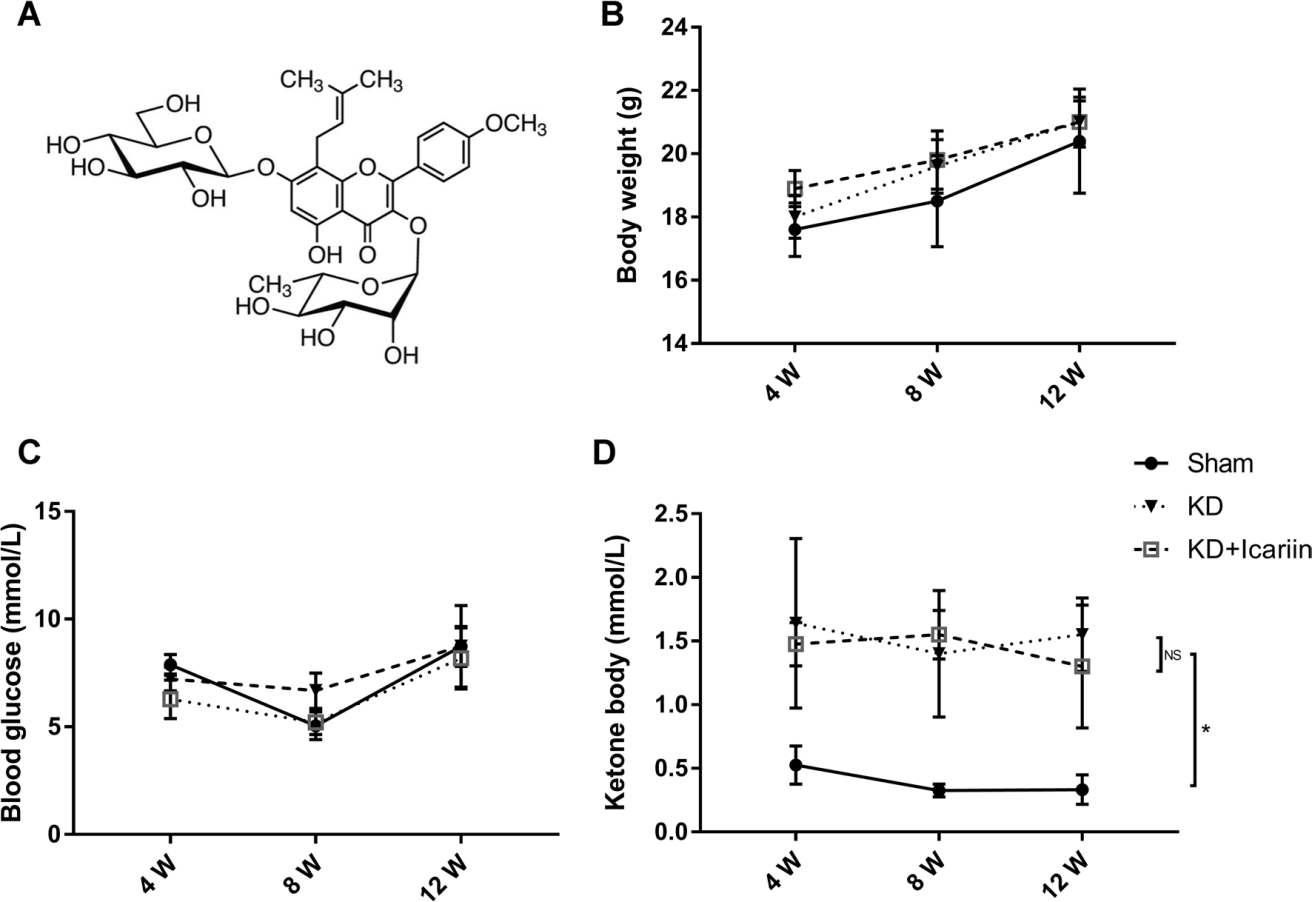
Broad indicators and chemical composition. **A** Structural formula of icariin. Comparison of body weight (**B**), blood glucose (**C**), and blood ketone levels (**D**) across different groups. No significant differences were observed in body weight and blood glucose among the sham, KD, and KD + icariin groups. However, ketone body levels were notably higher in these groups. Data were presented as means and standard deviations, with a *P < 0.05 difference compared to the sham group

Throughout the 12-week intervention, all groups exhibited weight gain, yet no significant differences were noted between them at any evaluation point (Fig. 1B; *P* > 0.05). Blood glucose levels remained consistent across the groups (Fig. 1C; *P* > 0.05). However, ketone body levels in blood of sham group were markedly lower than those in both the KD and KD + icariin groups (Fig. 1D; *P* < 0.05). These findings imply that while icariin exerted negligible effects on blood glucose and ketone bodies, the ketogenic diet effectively in raising blood ketone body levels in the mice.

### Icariin modifies markers of bone regeneration

**Fig. 2:**
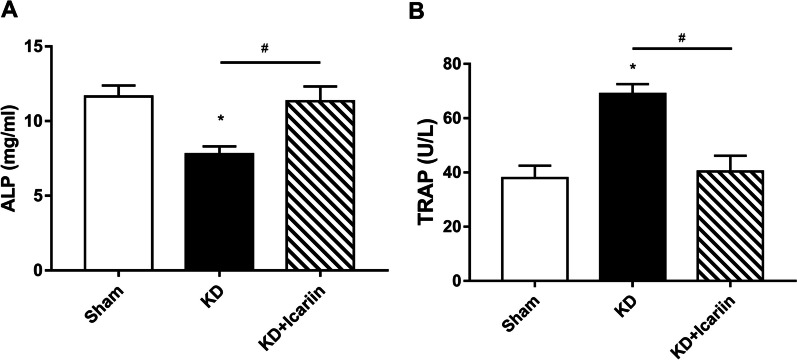
Serum indicators in various groups. Changes in serum concentrations of ALP (**A**) and TRAP (**B**). In the KD + icariin group, icariin effectively elevated the concentration of ALP which was significantly reduced by KD alone. TRAP levels, which were markedly increased in the KD group, were significantly reduced by icariin in the KD + icariin group. The results were presented as means with standard deviation, with *P < 0.05 compared to the sham group and #*P* < 0.05 between KD and KD + icariin

Osteoblast and osteoclast activities are represented by serum biochemical markers, specifically ALP and TRAP. ELISA data indicated that the ALP level in the KD group was significantly lower than that in the sham group (Fig. 2A; *P* < 0.05), while TRAP level higher (Fig. 2B; *P* < 0.05). Post-icariin treatment, ALP level in the KD + icariin group increased notably compared to the KD group (Fig. 2A; *P* < 0.05), approaching those of the sham group. Similarly, TRAP levels in the KD + icariin group were comparable to those in the sham group (Fig. 2B; *P* < 0.05).

### Icariin improves deterioration of bone microarchitecture

**Fig. 3:**
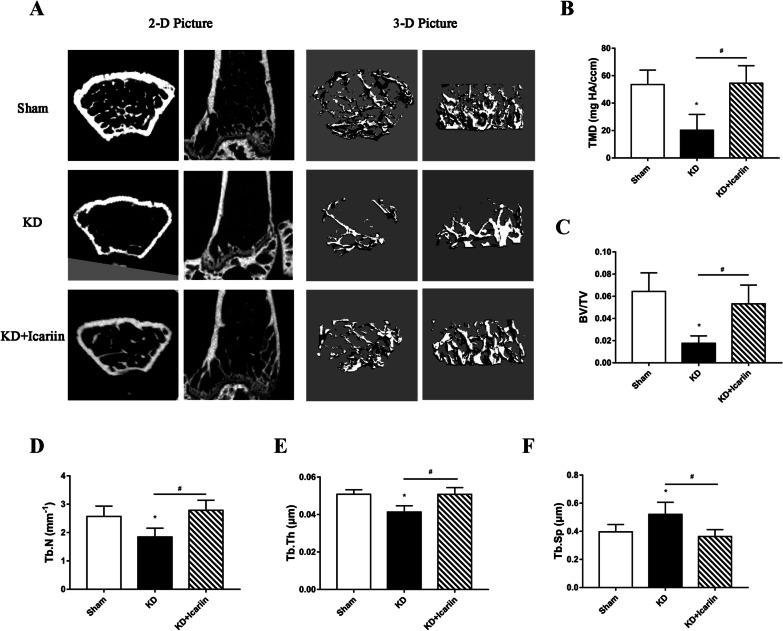
Microarchitecture of cancellous bone in the distal femur. **A** Three-dimensional and two-dimensional images of cancellous bone. The trabecula parameters of the cancellous bone are detailed in sections **B**, **C**, **D**, **E**, and **F**. In the KD + icariin mice, icariin effectively mitigated bone loss and maintained microarchitectures integrity. Data were presented as mean and standard deviation, with *P＜0.05 for comparison with the sham group and #*P*＜0.05 for comparison between the KD and K + icariin groups

Micro-CT analysis showed that the left distal femoral cancellous bone in the KD group had a deteriorated microstructures, with significant trabecular degradation and bone thinning (Fig. 3A). Parameters such as TMD, BV/TV, Tb.N, and Tb.Th were notably reduced, while Tb.Sp was increased in the KD group compared to the sham group (*P* < 0.05). However, icariin intervention markedly improved trabecular bone condition. In the KD + icariin group, there was a significant increase TMD, BV/TV, Tb.N, and Tb.Th, and a substantial reduction in Tb.Sp compared to the KD group (*P* < 0.05). The results demonstrated that icariin significantly mitigated bone loss and reversed the degradation of bone microstructures in KD mice.

### Icariin balances bone turnover markers in cancellous bone

**Fig. 4:**
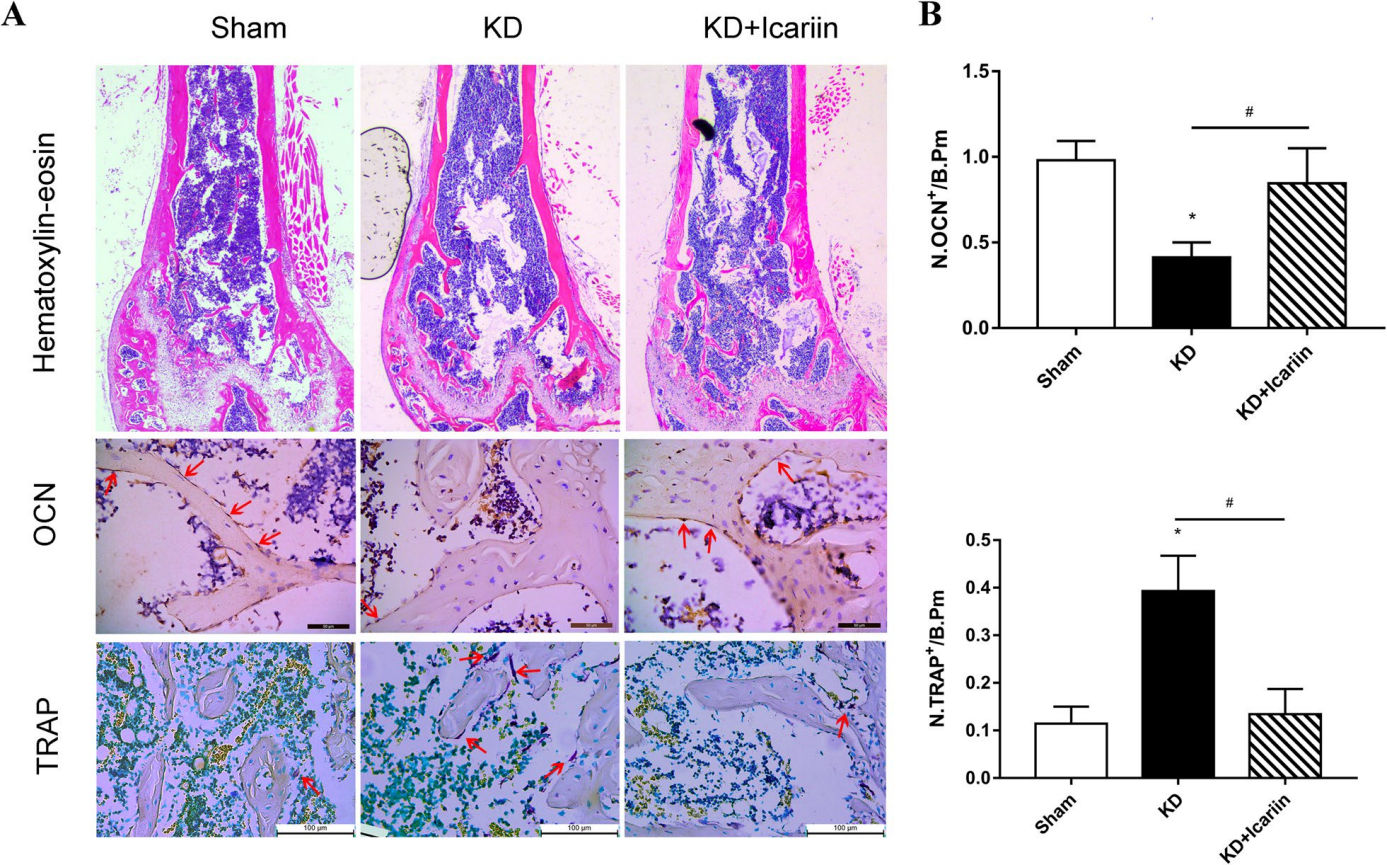
Immunohistochemical and histological staining. **A** Differences in distal femur hematoxylin–eosin, OCN, and TRAP staining of the distal femur among groups. Red arrows indicate positive osteoblast and osteoclast, respectively. **B** The density of OCN and TRAP positive cells on the bone surface was quantified as cells per millimeter of bone perimeter (/B.Pm). Data were presented as means and standard deviations, and *P < 0.05 was used to compare the study to a control group, and #*P* 0.05 was used to compare the KD and KD + icariin groups

Hematoxylin–eosin staining demonstrated significant microstructural degradation and a reduction trabecular bone in the KD group compared to the sham group. Icariin administration, however, effectively mitigated trabecular bone loss in the KD + icariin group (Fig. 4A). The KD group showed markedly lower OCN expression and higher TRAP expression than the sham group (Fig. 4; *P* < 0.05). Post-icariin treatment, an increase in osteoblasts expressing OCN and a decrease in osteoclasts expressing TRAP were observed compared to the KD group. Additionally, icariin significantly reduced tissue TRAP expression while enhancing tissue OCN expression (Fig. 4; *P* < 0.05).

### Icariin enhances BMSCs' osteogenic differentiation

**Fig. 5:**
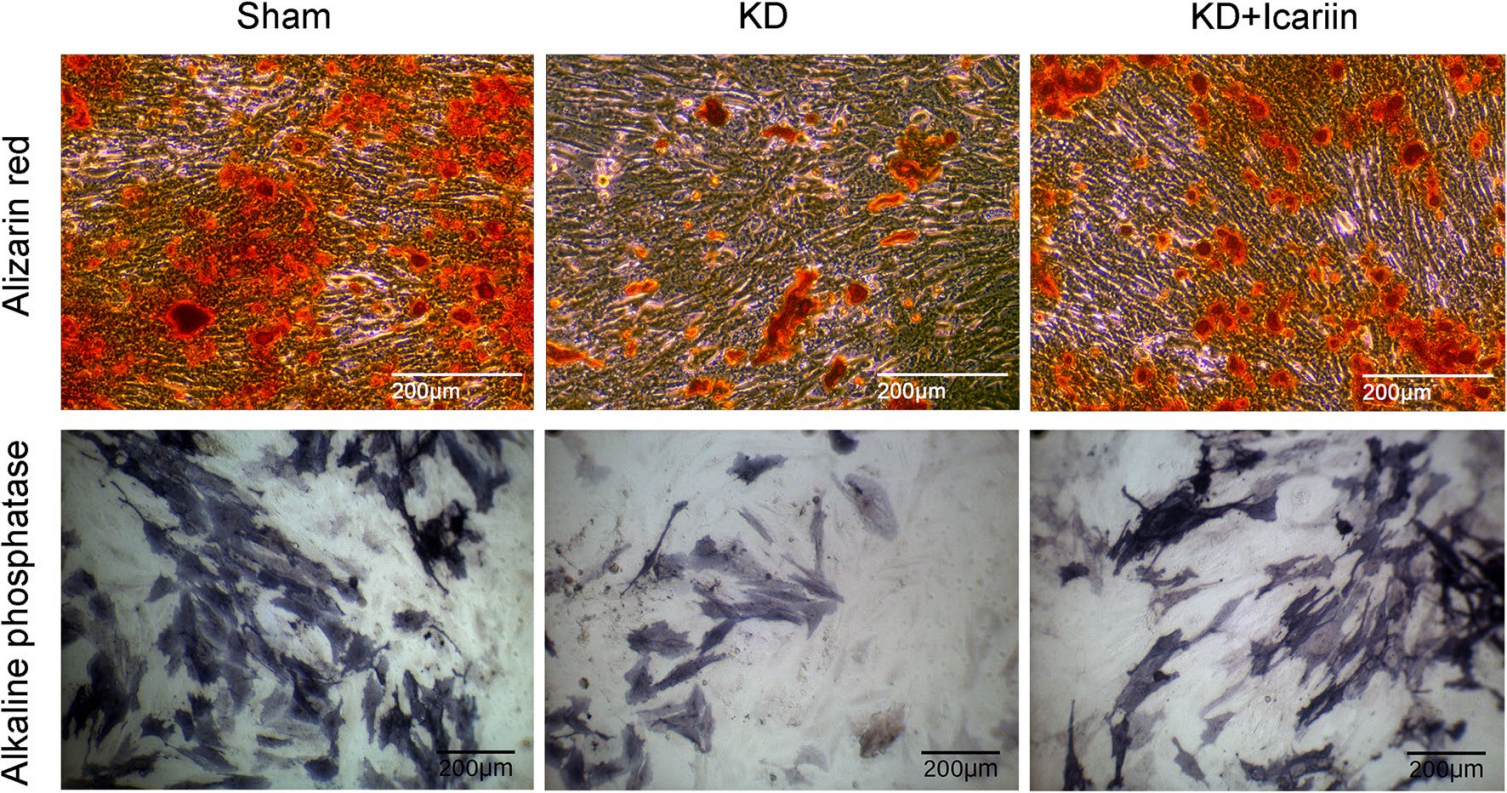
Alizarin red and cellular ALP staining. KD reduced calcium deposits and cellular ALP levels, which cells from the KD + icariin mice exhibited increased ALP levels and calcium salts following icariin intervention

Alizarin red and ALP staining, key indicators of BMSC osteogenic differentiation, showed that BMSCs from the KD group had diminished ALP levels and activity compared to the sham group. However, following icariin intervention, ALP levels and activity in the KD + icariin group improved, aligning with those of the sham group (Fig. 5). Alizarin red staining indicated a reduced calcium salt deposition in the KD group compared to the sham group. Post-icariin intervention, the KD + icariin group exhibited increased calcium salt deposition, akin to the sham group (Fig. 5). These results suggest that icariin enhances the osteogenic differentiation capacity of BMSCs following KD intervention.

### Icariin promotes osteogenesis through the mTOR/autophagy signaling pathway

**Fig. 6:**
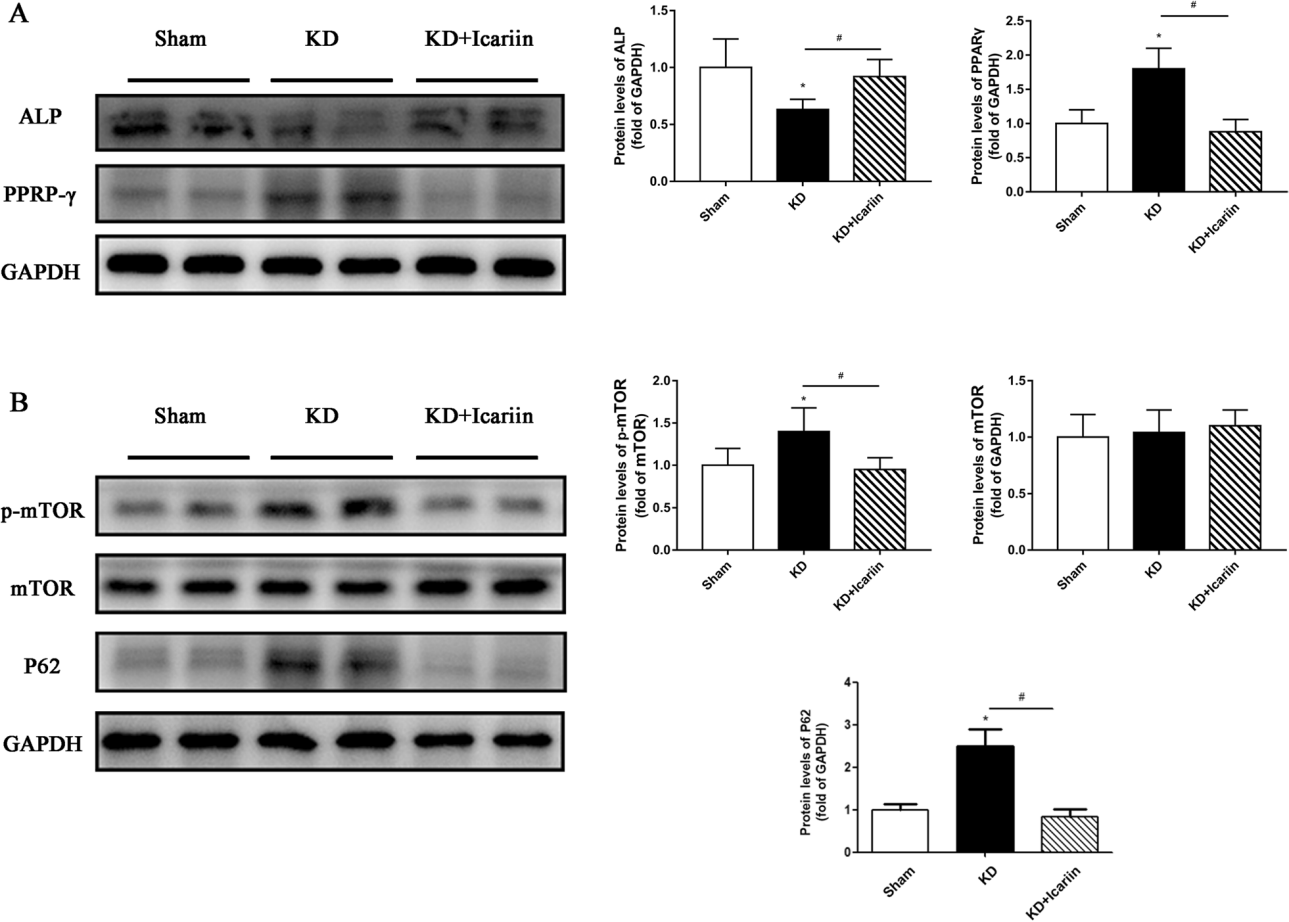
Protein expression of the distal femur. **A** Western blot bands illustrating the semiquantitative results for ALP and PPAR-γ. **B** Western blot bands and the semiquantitative findings for mTOR, p-mTOR and P62. In KD + icariin mice, icariin drastically reduced the expression of PPAR-γ, p-mTOR and P62, while significantly enhanced the expression of ALP. Data were presented as averages and standard deviations, with *P < 0.05 was used to compare the sham group with the control group and #*P* < 0.05 was used to compare the KD and KD + icariin groups

Western blot analysis revealed that in the KD group, PPAR-γ expression was elevated while ALP expression was reduced compared to the sham group (Fig. 6A; *P* < 0.05). In the KD + icariin group, there was a significant decrease in PPAR-γ expression and an increased in ALP expression compared to the KD group (*P* < 0.05). The levels of p62 and p-mTOR were significantly higher in the KD group than in the sham group (*P* < 0.05). However, icariin intervention led to a significant reduction in p62 and p-mTOR levels in the KD + icariin group compared to the KD group, while mTOR levels remained consistent across all three groups (Fig. 6B; *P* > 0.05). These findings suggested that icariin, under KD conditions, effectively promotes osteogenic differentiation of BMSCs might through the mTOR/autophagy signaling pathway.

## Discussion

Our previous research established that KD disturbs the balance between osteoblasts and osteoclasts, leading to osteoporotic syndrome [25]. In this study, our objective was to explore whether icariin influences the differentiation of BMSCs into osteogenic and adipogenic cells by modulating the mTOR/autophagy pathway, and to assess its effects on body weight, blood biochemical indicators, bone mass, and bone turnover biomarkers in the context of KD. The results indicated that icariin effectively mitigates bone loss in the KD group, enhances osteogenic differentiation via the mTOR/autophagy pathway, strengthens osteoblast activity by impeding adipogenic differentiation, significantly reduces the number of osteoclasts, and restores the bone microstructures.

Decreased bone mass and trabecular bone destruction are primary contributors to osteoporosis, making bone microstructure is a crucial metric for assessing bone loss [26]. Bone remodeling encompasses the removal of old bone (bone resorption) and the formation of new bone (bone formation) [27]. The process involves continuous bone resorption by osteoclasts and subsequent replacement by osteoblasts, which produce new bone [28]. Bone turnover markers are specific biological products indicative of bone resorption and formation processes. ALP and OCN are rare markers of osteoblast activity, while TRAP is used to gauge osteoclasts numbers [29]. In this study, KD intervention led to a significant decline in serum ALP levels, a substantial decrease in cancellous bone tissue OCN expression, and a marked increase in serum and cancellous bone tissue TRAP levels, indicating that KD impairs bone microstructure and contributes to osteoporosis.

Furthermore, BMSCs undergo two pivotal differentiation pathways: osteogenic and adipogenic differentiation. An imbalanced between these processes can disrupt bone remodeling [30]. The sustained and specific expression of ALP is crucial for osteogenic differentiation, aiding the transformation of BMSCs into osteoblasts, which predominantly secrete OCN, indicative of new bone formation [31]. Adipogenic differentiation, occurring simultaneously with osteogenic differentiation, is primarily driven by PPAR-γ, a key factor in the evolution of BMSC into adipocytes [32]. An increased adipogenic differentiation, which contributes to osteoporosis, is often accompanied by a decrease in osteogenic differentiation. Our research revealed that under KD condition, osteogenic differentiation of BMSCs was reduced, leading to osteoporosis, as evidenced by decreased ALP expression and increased PPAR-γ expression in the KD group.

Icariin, the main pharmacologically active compound, the traditional Chinese medication Epimedium, has demonstrated extensive clinical therapeutic potential [33]. Recent studies have focused on icariin bolsters the osteogenic differentiation of BMSCs, stimulates osteoblast proliferation, and exhibited anti-osteoporotic properties through the enhancement of intracellular osteoblast-related gene expression [34]. It has shown efficacy in reducing osteoporotic symptom in rats with ovariectomies, significantly improving bone healing and remodeling in osteoporotic fracture models, and suppressing TRAP expression while increasing ALP activity and decreasing osteoclast activity. These outcomes corroborate previous findings [21, 22]. In this study, icariin improved TMD, BV/TV, Tb.N, and Tb.Th while reducing Tb.Sp in the bone microstructures under KD intervention. Additionally, it markedly elevated serum ALP levels, increased cancellous bone tissue OCN expression, and lower serum and cancellous bone tissue TRAP levels. Moreover, icariin augmented ALP in serum, boosted OCN and ALP protein expression in tissues, and suppressed PPAR-γ in tissues, thereby facilitating BMSCs differentiation. Hence, icariin not only prevents the deterioration of bone microstructures but also maintains bone turnover balance, promoting osteogenic differentiation of BMSCs and inhibits adipogenic cells development.

The process of bone remodeling encompasses autophagy, where its overexpression fosters BMSCs differentiation, enhances osteoblast function, and even protects osteoblasts from apoptosis. Osteoporosis occurs when autophagic activity diminishes due to aging, reduced BMSCs and osteoblast development, increased apoptosis, loss of bone mass, and deterioration of the bone microstructures [17]. The signaling pathway regulating autophagy in bone acts as a critical nexus for cascade signaling to downstream autophagy proteins, with mTOR being a key component. Activation of mTOR signaling, which may promote p62 expression, influences the ability of BMSCs and osteoblasts to form bone, differentiate into adipogenic cell, and maintain osteocytes homeostasis [35]. In this study, KD intervention increased p-mTOR and p62 expression, while icariin + KD combination reduced them. Moreover, KD notably impaired osteoblast differentiation in cellular assays, but icariin treatment subsequently enhanced the BMSCs’ development into osteoblasts. In summary, the data indicate that icariin may bolster anti-osteoporotic effects in mice by stimulating the mTOR/autophagy pathway.

Autophagy is integral to the overall process of bone metabolism, involving osteoblasts, osteocytes, osteoclasts, and BMSCs. Elevated autophagic activity helps beneficial for cells survival and functionality. While decreased autophagic activity is linked to osteoporosis, increased activity also promotes differentiation in both osteogenic and osteoclastic cells [17]. A limitation of this work is the absence of osteoclast differentiation assessment in cellular experiments, as these two cell types mutually regulate and balance each other. Secondly, molecules such as PI3K/AKT [36] upstream and ULK1 [37] downstream were utilized to gauge the activity of mTOR, a pivotal regulator of bone-associated autophagy. This study solely relied on contingency as an index, while mTOR's downstream products reveal specific impacts on osteoblasts and osteoclasts, as well as the link between osteogenic differentiation and osteoclastic differentiation. Additionally, this study focused only on the protective effect of icariin on cancellous bone, omitting other significant bone formation and resorption markers like cathepsin K, typically present in bone tissue. Future research is needed to elucidate the underlying mechanism.

## Conclusions

Icariin demonstrates efficacy in enhancing bone quality, reducing bone loss, and treating osteoporosis induced by KD. It accomplished this by inhibiting mTOR and promoting autophagy, thus facilitating osteogenic differentiation of BMSCs. Consequently, these findings lay a theoretical groundwork and offer guidance for the clinical application of icariin in bone metabolism, particularly in treating KD-induced osteoporosis.

## Data Availability

All data generated or analyzed during this study are included in this published article.

## Abbreviations

ALP: Bone-specific alkaline phosphatase
BMSCs: Bone marrow mesenchymal stem cells
BV/TV: Bone volume fraction
H and E: Hematoxylin and eosin staining
KD: Ketogenic diet
mTOR: Mammalian target of rapamycin
OCN: Osteocalcin
PPAR-γ: Peroxisome proliferator-activated receptor gamma
Tb.N: Trabecular number
Tb.Th: Trabecular thickness
Tb.Sp: Trabecular separation
TMD: Bone tissue density
TRAP: Tartrate-resistant acid phosphatase
